# Association of dietary inflammatory index with constipation: Evidence from the National Health and Nutrition Examination Survey

**DOI:** 10.1002/fsn3.3914

**Published:** 2024-01-17

**Authors:** Bo Wang, Chunxiang Liu, Zheng Guo, Rui Li, Yuchao Wang, Caixia Dong, Daqing Sun

**Affiliations:** ^1^ Department of Paediatric Surgery Tianjin Medical University General Hospital Tianjin China; ^2^ Tianjin Key Laboratory on Technologies Enabling Development of Clinical Therapeutics and Diagnosis, School of Pharmacy Tianjin Medical University Tianjin China

**Keywords:** constipation, cross‐sectional study, dietary inflammatory index, NHANES

## Abstract

The association of dietary inflammatory index (DII) with constipation has not been well studied in general population. Therefore, the aim of this cross‐sectional study was to investigate whether DII is associated with constipation in a large representative sample of the US population. Data were obtained from the 2005–2010 National Health and Nutrition Examination Survey (NHANES). A total of 12,308 participants aged ≥20 years were included in the analysis. DII was calculated based on a single 24‐h dietary recall, and constipation was defined as having fewer than three bowel movements per week by the questionnaire on bowel health. Logistic regression analysis demonstrated a significant positive association between DII score and constipation, with each unit increase in DII score associated with a 20% increase in constipation risk (95% CI: 1.13–1.28). Subgroup analysis revealed high odds ratios (ORs) among individuals classified as “Other Race” (OR: 1.42, 95% CI: 1.12–1.80) and “Non‐Hispanic White” (OR: 1.31, 95% CI: 1.12–1.54). In addition, RCS analysis indicated a nonlinear relationship between DII and constipation among individuals with a BMI less than 25 (OR: 1.17, 95% CI: 1.07–1.28), while the overall trend remained positive correlation (OR: 1.20, 95% CI: 1.10–1.31). Briefly, our study suggests that there may be a link between DII and constipation, which has implications for the development of dietary interventions aimed at preventing and managing constipation. However, this association was complex and variable depending on individual factors such as BMI and racial background and needed to establish longitudinal studies to confirm the underlying mechanisms between DII and constipation.

## Graphical abstract

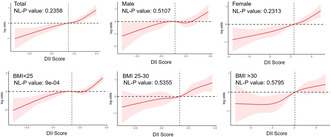

## 1 INTRODUCTION

Constipation, an ailment that involves decreasing stool frequency and difficult defecation, is a common gastrointestinal disorder affecting approximately 20% of the general population (Chen et al., 2019; Dalziel et al., 2017). Notably, constipation is often accompanied by bloating, abdominal pain, and a sense of unease (Bielefeldt et al., 2016). The etiology of digestive diseases is multifaceted, with factors such as low fiber intake, dehydration, adverse health behaviors, such as smoking, excessive alcohol consumption, and a lack of physical activity, all contributing to its development (Szymaszkiewicz et al., 2022). Moreover, depression can potentially impact the neuroendocrine system, consequently influencing gastrointestinal functions and leading to the onset or exacerbation of constipation (Wang et al., 2023). Recent studies have suggested that chronic inflammation, a natural response of the immune system to injury or infection, may also play a role in the development of constipation (Furman et al., 2019; Ju et al., 2020). However, when inflammation becomes chronic, it promotes a series of diseases such as diabetes, cardiovascular disease, and cancer (Denova‐Gutiérrez et al., 2018).

To measure the inflammatory potential of one's diet, the dietary inflammatory index (DII) is utilized. It assesses the impact of various foods and nutrients on the inflammatory potential of an individual's diet (Kotemori et al., 2021). Previous studies have highlighted that a higher DII score may increase the risk of various chronic diseases (Cha et al., 2021). The relationship between DII score and constipation, however, remains elusive. To explore the possible association between a higher dietary inflammatory index and an increased incidence of constipation, our study aims to identify potential dietary interventions to help alleviate this common condition. Unhealthy eating habit is closely related to constipation since its influence on inflammation. For instance, the Western dietary pattern, which is loaded with meat, dairy products, fat, sugary foods, processed meats, pastries, sweetened drinks, alcohol, and limited amounts of vegetables and fruits has been linked to an increased presence of inflammatory markers in the body, and alter the intestinal microbiota, promoting a low‐grade chronic inflammation in the gut. Correspondingly, anti‐inflammatory diets could reduce this level, adherence to the anti‐inflammatory dietary pattern shows a beneficial effect on the gut microbiome and gut metabolites (Tayyem et al., 2021). Notably, studies conducted on humans have demonstrated a link between a deficiency in dietary fiber and persistent constipation (Gill et al., 2021). In fact, persistent constipation is recognized as the most prevalent digestive symptom of diabetes (Bharucha et al., 2013). Dietary patterns, which are subject to change, also have a profound association with the onset of chronic constipation. In regular clinical practice, dietary management is widely regarded as the cornerstone of any therapy for persistent constipation (Yang et al., 2022). Nevertheless, due to sample size and research methodology limitations, this study primarily focuses on a narrow subset of food constituents, such as fiber and energy intake, which may not fully capture the complexity of the human diet and its relationship with constipation.

In order to deepen our comprehension of the correlation between diet and inflammation in constipation, we performed a cross‐sectional study using the National Health and Nutrition Examination Survey (NHANES) database. Our primary objective was to investigate the potential association between DII and constipation, while controlling for potential confounding variables, such as age, sex, socioeconomic status, and other dietary factors. The NHANES database is a valuable resource, as it provides population‐representative data on various health indicators at a national level (Park et al., 2020). Through our findings, we aim to provide meaningful insights that may inform the development of more effective dietary interventions for individuals with chronic constipation.

## 2 MATERIALS AND METHODS

### 2.1 Study design and participants

The NHANES database is a nationally representative cross‐sectional survey program that aims to evaluate the health and nutritional status of the noninstitutionalized population in the United States. The sampling method used is complex and involves several stages of probability clustering, stratification, and clustering. The Ethics Review Board of the National Center for Health Statistics (NCHS) approved the protocol, and all participants provided written informed consent. We utilized data from the NHANES 2005–2010 cycles, as they were the only cycles that included information on both bowel habits and dietary intake. Participants who lacked comprehensive data on their bowel habits, calorie consumption, dietary fiber intake, smoking, and drinking results were excluded from the study. Although the NHANES survey was not specifically designed to investigate digestive disorders, data on constipation were extracted from a set of broad questions, such as the Bowel Health Questionnaire. Access to data and samples from the NHANES is publicly available for further research.

### 2.2 Definition of constipation

Defining constipation based on stool frequency has been considered a validated metric used by previous research that evaluated constipation in NHANES surveys. Three NHANES cycles from 2005 to 2010 included a question about stool frequency, asking participants “How often have you had a bowel movement (BM) each week over the past 30 days?” The range of possible responses was not specified, but it ranged from one BM to 70 BMs per week. For this investigation, chronic constipation was defined as having less than three BMs per week.

### 2.3 Dietary inflammatory index score

The DII is a scoring system that Shivappa developed to evaluate the potential inflammatory effects of various nutrients based on a comprehensive review of scientific literature. This scoring system considers both proinflammatory and anti‐inflammatory properties of the diet by assessing the intake of 45 different nutrients. To calculate the DII score, the intake of each dietary component over the previous 24 h is evaluated, and the *Z* score is determined. This *Z* score is subsequently converted into a percentile score and multiplied by 2, then 1 is subtracted to achieve a symmetrical distribution. The individual scores were summed to produce an overall DII score, a valuable tool for assessing the inflammatory potential of the diet. By using a comprehensive approach evaluates multiple dietary components, the DII provides a more accurate and nuanced picture of the inflammatory potential of the diet. It offers healthcare professionals more effective dietary interventions to improve overall health outcomes, gaining better insights into the complex relationship between diet and inflammation.

In our study, the DII score ranged from −5.28 to 5.41, and quartiles were established based on the distribution of scores. The lowest dietary inflammatory group was designated as Q1 (−5.28 ≤ DII < −0.34), followed by Q2 (−0.34 ≤ DII <1.85), Q3 (1.85 ≤ DII <3.03), and the highest dietary inflammatory group in Q4 (3.03 ≤ DII <5.41). It was noteworthy that the DII score was still calculated even if the number of nutrients used for its calculation was less than 30. Establishing quartiles based on the DII score distribution provides a more nuanced analysis of the relationship between dietary inflammation and constipation. This enables us to investigate potential dose–response relationships, adding greater rigor to the study's findings. Furthermore, less comprehensive dietary data by using fewer than 30 nutrients were included for DII calculation added flexibility and offered valuable insights into the association between diet and constipation. Overall, the use of the DII score and the establishment of quartiles provides more comprehensive approach to the study of the relationship between diet and constipation.

### 2.4 Additional variables

In the study, we collected and analyzed several variables in addition to DII and constipation. Firstly, demographic information such as age, gender, race/ethnicity, education, and marital status was included in our study. Next, the impact of lifestyle factors such as smoking status and alcohol consumption, and medical history including a history of diabetes, hypertension, and other conditions, which were obtained through self‐reported questionnaires, were also recorded. Subsequently, body mass index (BMI) and blood pressure were measured and recorded during physical examinations. BMI was classified into three categories: <25 kg/m^2^, 25 kg/m^2^ to 30 kg/m^2^, and ≥30 kg/m^2^. In addition, a Patient Health Questionnaire (PHQ‐9) was also included to measure the severity of depression in individuals. The PHQ‐9 score was categorized into four groups: 0–4 represented the absence of depression, 5–9 indicated mild depression, 10–14 suggested moderate depression, and a score of 15 or higher was considered as moderately severe to severe depression. Validated cutoff scores for depression severity were used in our study. The dietary intake of fiber (in grams), water (in grams), and energy (in kilocalories) from the total nutrient file were obtained. The file was generated by summing up the nutrients consumed by each participant from all reported foods and beverages in the dietary recall.

### 2.5 Statistical methods

In our study, we utilized a comprehensive data analysis approach, taking into account the weighted and clustered sample design recommended by NHANES. We incorporated the newly reweighted 6‐year weight, which accounted for 1/3 of the weight from 2005 to 2010, in order to obtain more accurate results. Categorical variables were presented as percentages with 95% confidence intervals (CIs), allowing for a more nuanced understanding of the data. We also expressed continuous variables as mean standard error, which provided a more complete sight of the data distribution. To assess the relationship between DII and constipation, we performed logistic regressions, taking into account a wide range of factors that could influence the outcome. We first treated DII as a continuous variable and then categorized it into quartiles, with the lowest quartile serving as the reference group, providing a more granular analysis of the data. Our results were reported as odds ratios (OR) and CI, which allowed for a more precise interpretation of the findings. We also made four hierarchical adjustments in the logistic regressions, including age, gender, racial and ethnic origin, educational level, marital status, diabetes, blood pressure, smoking, drinking, depression, BMI, and dietary intake, such as calories and fiber, to account for a broad range of factors that could influence the outcome. To further investigate the relationship between DII and constipation, we conducted subgroup and interaction analyses for age group, gender, BMI, race, diabetes, depression, and hypertension in fully adjusted logistic regressions. This allowed for a more in‐depth examination of the data, taking into account individual differences that could affect the outcome. Additionally, we used restricted cubic splines (RCS) with four knots in each logistic regression model to test the nonlinear relationship between DII and constipation. Overall *p* and nonlinear *p* values in RCS were obtained by Wald tests using the “ANOVA” programming command in R, providing a more nuanced understanding of the data.

## 3 RESULTS

### 3.1 Characteristics of the study sample

**FIGURE 1:**
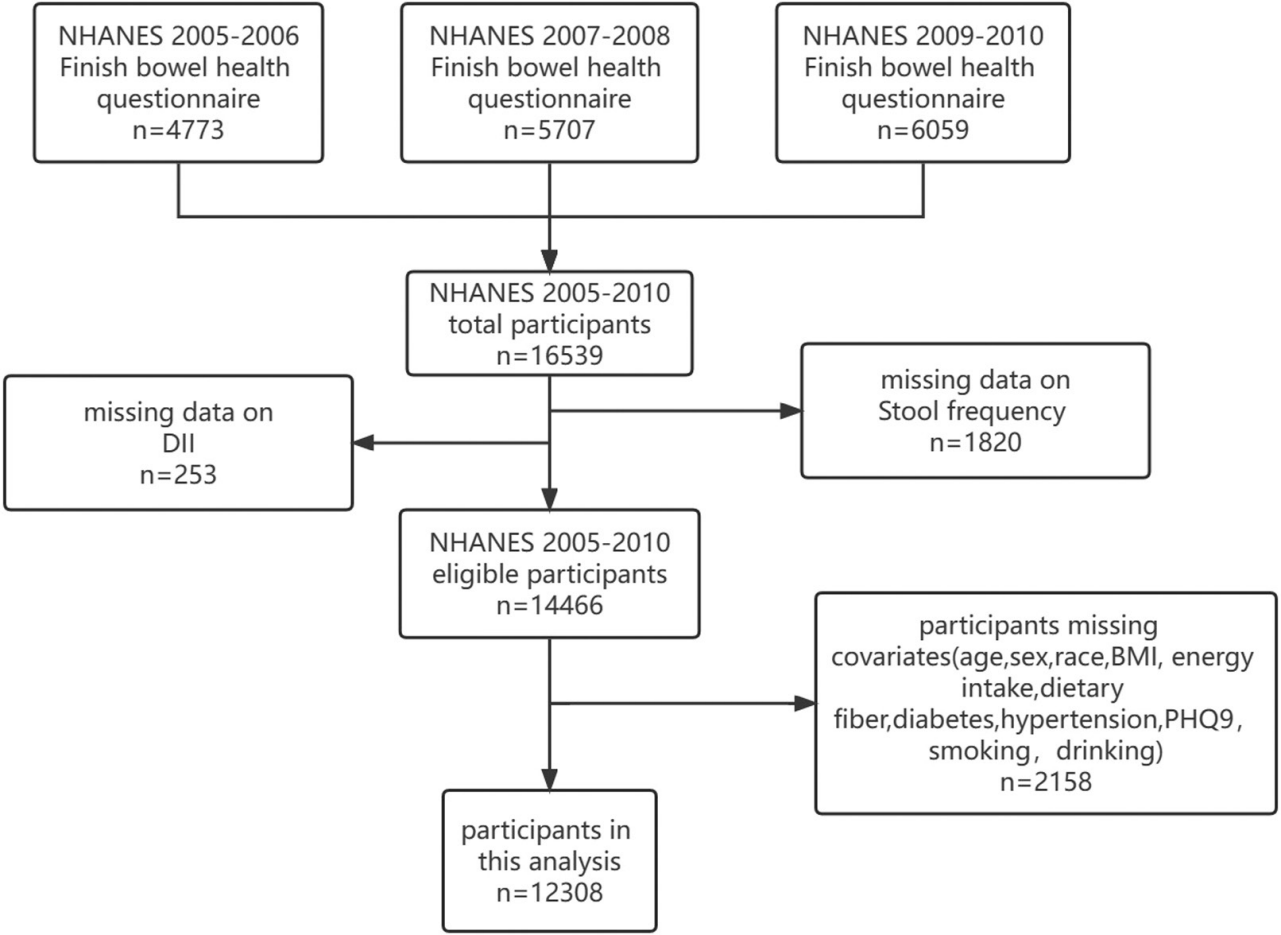
The flowchart illustrates the process of sample selection from NHANES 2005–2010.

We used the following criteria for participant exclusion: (1) missing data on the Bowel Health Questionnaire and DII, and (2) lack of information on covariates, including age, race, gender, level of education, marital status, diabetes, hypertension, BMI, depression, and nutritional consumption, such as energy and fiber (details on inclusion/exclusion criteria are presented in Figure 1). Ultimately, 12,308 participants were enrolled in the study. Table 1 compares individuals without constipation (*N* = 11,322) and constipation (*N* = 986) in terms of various demographic, lifestyle, health‐related factors, and dietary habits. The results indicate that individuals with constipation are more likely to be female, non‐Hispanic Black, living alone, and have lower levels of education. They are also more likely to be former smokers and have a higher prevalence of diabetes and depression. In terms of dietary habits, individuals with constipation have a lower intake of fiber and water, and a higher DII score than those without constipation. These findings suggest that lifestyle and dietary factors may play a significant role in the development of constipation and should be considered in its prevention and management.

**TABLE 1:** The weighted baseline characteristics of the participants.

	No constipation (*N* = 11,322)	Constipation (*N* = 986)	*p*‐Value
Age (years)	47.82 (0.36)	44.22 (0.68)	<.001
Sex (%)			
Female	5521 (48.8%)	701 (71.1%)	<.001
Male	5801 (51.2%)	285 (28.9%)	
Race (%)			
Mexican American	2030 (17.9%)	99 (10.0%)	<.001
Non‐Hispanic Black	2118 (18.7%)	292 (29.6%)	
Non‐Hispanic White	5795 (51.2%)	490 (49.7%)	
Other Race	1379 (12.2%)	105 (10.6%)	
Marital status (%)			
Living alone	4246 (37.5%)	446 (45.2%)	<.001
Married or living with partner	7069 (62.4%)	540 (54.8%)	
Unknown	7 (0.1%)	0 (0%)	
Education level (%)			
Above high school	5597 (49.4%)	441 (44.7%)	.021
Below high school	3014 (26.6%)	272 (27.6%)	
High school	2701 (23.9%)	272 (27.6%)	
Unknown	10 (0.1%)	1 (0.1%)	
Smoking status (%)			
Former	3042 (26.9%)	188 (19.1%)	<.001
Never	5906 (52.2%)	540 (54.8%)	
Now	2374 (21.0%)	258 (26.2%)	
Drinking status (%)			
Former	2285 (20.2%)	225 (22.8%)	.002
Heavy	2228 (19.7%)	208 (21.1%)	
Mild	3680 (32.5%)	271 (27.5%)	
Moderate	1671 (14.8%)	131 (13.3%)	
Never	1458 (12.9%)	151 (15.3%)	
Diabetes (%)			
No	9555 (84.4%)	857 (86.9%)	.039
Yes	1767 (15.6%)	129 (13.1%)	
Hypertension (%)			
No	6454 (57.0%)	603 (61.2%)	.012
Yes	4868 (43.0%)	383 (38.8%)	
BMI (kg/m^2^) (%)			
<25 kg/m^2^	3091 (27.3%)	361 (36.6%)	<.001
>30 kg/m^2^	4299 (38.0%)	317 (32.2%)	
25–30 kg/m^2^	3932 (34.7%)	308 (31.2%)	
Depression severity (%)			
None	8760 (77.4%)	637 (64.6%)	<.001
Mild	1665 (14.7%)	186 (18.9%)	
Moderate	570 (5.0%)	98 (9.9%)	
Severe	327 (2.9%)	65 (6.6%)	
Energy intake (kal/day)	2066.37 (14.94)	1846.28 (30.30)	<.001
Fiber intake (gm/day)	17.04 (0.17)	13.30 (0.34)	<.001
Water intake (gm/day)	590.12 (17.96)	410.58 (28.00)	<.001
DII	1.35 (0.04)	2.26 (0.06)	<.001

### 3.2 The positive association between DII and constipation

**FIGURE 2:**
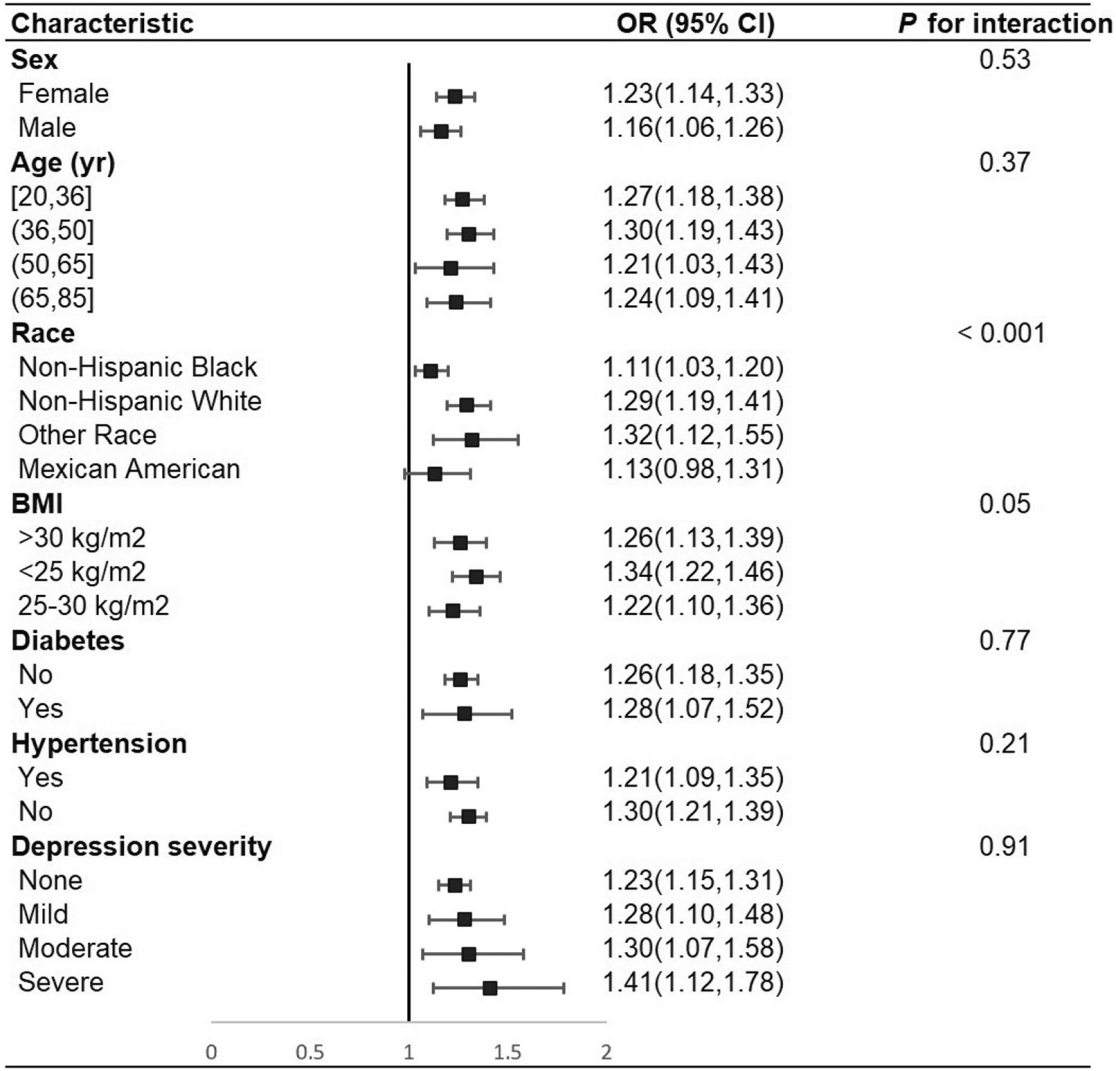
Forest plot displaying the associations between DII and constipation stratified by relevant variables.

**FIGURE 3:**
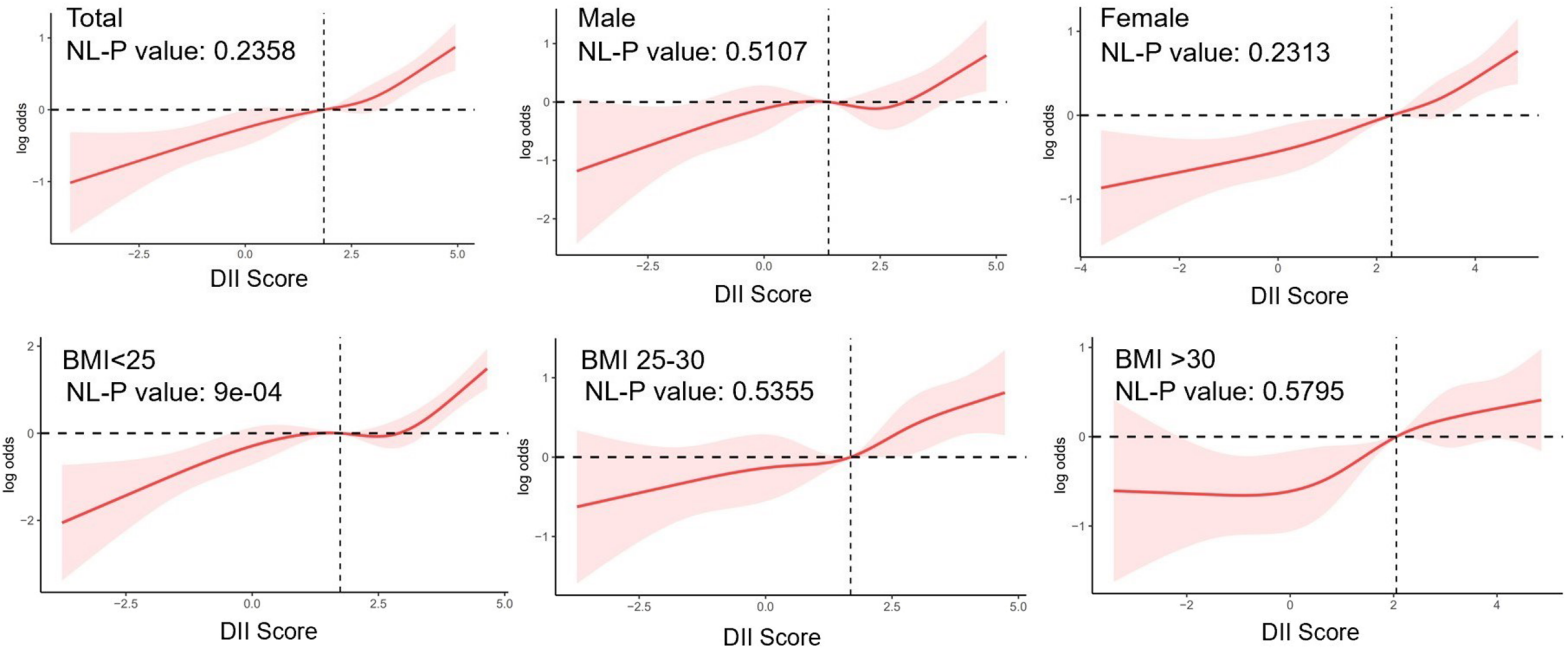
The relationship between DII and constipation in various groups was visualized using restricted cubic splines (RCS). The solid red line represents the odds ratio (OR) of constipation relative to DII, with the 95% confidence interval (95% CI) shown in pink.

Table 2 presents the results of a logistic regression analysis examining the association between DII score and constipation. DII score was analyzed as both a continuous variable and a categorical variable divided into quartiles (Q1–Q4). The analysis involved several models adjusted for different covariates. The results show that there was a significant positive association between DII score and constipation, meaning that increasing DII score was associated with increasing constipation risk. In the final model, each unit increase in DII score was associated with a 20% increase in constipation risk (95% CI: 1.13–1.28). Additionally, the analysis by quartiles reveals a dose–response relationship between DII score and constipation risk, where higher DII scores are associated with a higher risk of constipation (*p* for trend <.001). The results of the subgroup analysis are presented in Figure 2. The analysis reveals significant heterogeneity in the association between DII and constipation across different racial groups, with higher odds ratios (ORs) found among individuals classified as “Other Race” and “Non‐Hispanic White.” Conversely, other subgroups such as sex, age, hypertension, diabetes, and depression groups did not display a significant association between DII and constipation (*p* for interaction >.05). The results of RCS (Figure 3) analysis showed that the odds of psoriasis displayed a slightly increasing trend with increasing DII, there was no nonlinear association observed between DII and constipation. Moreover, it is noteworthy that a nonlinear relationship was observed in individuals with a BMI less than 25; however, the overall trend remained positive correlation.

**TABLE 2:** The findings from a logistic regression analysis investigating the relationship between DII score and constipation.

	Crude Model	Model 1	Model 2	Model 3
DII (continuous)	1.36 (1.30,1.42)	1.25 (1.19, 1.32)	1.25 (1.19, 1.31)	1.20 (1.13, 1.28)
DII (categories)				
Q1	Ref.	Ref.	Ref.	Ref.
Q2	1.58 (1.21,2.05)	1.38 (1.06, 1.81)	1.41 (1.07, 1.85)	1.27 (0.95, 1.69)
Q3	2.14 (1.62,2.84)	1.67 (1.25, 2.23)	1.68 (1.25, 2.26)	1.42 (1.03, 1.97)
Q4	3.72 (3.00,4.60)	2.55 (2.01, 3.24)	2.54 (1.98, 3.24)	2.03 (1.51, 2.74)
*p* for trend	<.001	<.001	<.001	<.001

*Note*: Data are presented as OR [95% CI]. The DII quartiles were established based on the distribution of scores. The lowest dietary inflammatory group was designated as Q1 (−5.28 ≤ DII < −0.34), followed by Q2 (−0.34 ≤ DII <1.85), Q3 (1.85 ≤ DII < 3.03), and the highest dietary inflammatory group in Q4 (3.03 ≤ DII < 5.41). Model 1 adjusted for age, sex, marital status, education levels, and race. Model 2 adjusted for age, sex, marital status, education levels, race, smoking, alcohol users, diabetes, depression severity, hypertension, and BMI. Model 3 adjusted for age, sex, marital status, education levels, race, smoking, alcohol use, diabetes, depression severity, hypertension, BMI, energy intake, fiber intake, and water intake. The following acronyms are used in the study: OR (odds ratio), CI (confidence interval), DII (Dietary Inflammatory Index), Q1 (first quartile), Q2 (second quartile), Q3 (third quartile), and Q4 (fourth quartile).

## 4 DISCUSSION

In this study, we aimed to elucidate the relationship between the DII and constipation within a comprehensive and representative sample of the US population. Our findings indicated that although there may be an association between DII and constipation, this relationship was not consistent across distinct racial groups. Although the odds of constipation exhibited an increase with higher DII scores, the association was not significant in either an overall or nonlinear context. Interestingly, a nonlinear association between DII and constipation was observed in individuals with a BMI below 25. These findings suggest that the relationship between DII and constipation was more intricate exceeded expectation, and more influenced by factors such as BMI and racial background.

The Western dietary pattern has been implicated in the development of hyperlipidemia, hyperglycemia, activation of nonenzymatic glycosylation, and glucose‐induced NADH, leading to excessive reactive oxygen species (ROS) generation, oxidative stress, and heightened systemic inflammation levels (Nickerson et al., 2014). Recent studies suggest that Western dietary patterns can disrupt the gut microbial ecosystem and cause chronic inflammation in the intestine (Wu et al., 2022). Additionally, a meta‐analysis of 22 high‐quality randomized clinical trials found that diet significantly affects blood inflammatory markers, with the Western diet increasing levels of IL‐1β, IL‐6, and CRP, while the Mediterranean diet demonstrates anti‐inflammatory properties (Bonaccio et al., 2017; Naja et al., 2017). The inflammatory potential of an individual's diet has been strongly correlated with various chronic diseases, unhealthy eating habits could activate the inflammasome complex, which plays a crucial role in gut disease pathology (Maiuolo et al., 2021). Numerous studies have demonstrated that intestines of constipated mice model display pathological changes, including the infiltration of inflammatory factors such as CD45 and TNF‐α in the intestinal mucosa and muscular layer. Proinflammatory diets unquestionably contribute to the accumulation of inflammatory factors within the body.

Our study suggested that a diet with a lower inflammatory potential, as measured by a lower DII score, may be beneficial for individuals with constipation. Modifying the diet to include more anti‐inflammatory foods and fewer proinflammatory foods may improve constipation. Examples of anti‐inflammatory foods include fruits, vegetables, whole grains, nuts, and fatty fish (Geng et al., 2020), while proinflammatory foods include processed and fried foods, sugary drinks, and red meat (Sreeja et al., 2019). Adding high‐fiber foods to the diet (Yang et al., 2016) could improve constipation (Oliveira et al., 2020). Drinking plenty of water and staying hydrated can also help soften stools and make them easier to pass. Fermented foods, such as yogurt and kefir, may also be beneficial for constipation, as it contains probiotics that can improve gut health (Companys et al., 2020; Yi et al., 2022). Some herbs and spices, such as ginger and turmeric, have anti‐inflammatory properties and may alleviate constipation (Ghasemian et al., 2016; Wei et al., 2017). Conversely, low‐fiber foods, such as processed and fried foods, sugary drinks, and red meat, can slow down digestion and make stools harder to pass, and consuming too much dairy or caffeine may also contribute to constipation in some individuals (Forootan et al., 2018). It is important to note that everyone's digestive system is different, and what worsens constipation for one person may not affect another person in the same way. Therefore, it is essential to pay attention to how different foods affect your body and adjust your diet accordingly. However, it is important to consult a healthcare professional before making any significant changes to the diet.

A recent cross‐sectional study found a potential association between the inflammatory potential of the diet, measured by the DII, and gut microbiota in individuals experiencing functional constipation. However, this study's limited sample size and focus on middle‐aged participants warrant further investigation to examine the relationship between DII and constipation in a more extensive and diverse population (Costa et al., 2022).

Our study bolsters the existing body of research connecting constipation to inflammation, as evidenced by the positive association between constipation and DII. Intriguingly, recent studies have identified a positive correlation between elevated DII scores and the onset of depression (You et al., 2022), which is often associated with gastrointestinal symptoms such as constipation and abdominal pain (Zhang et al., 2021). Consequently, enhancing dietary quality may prove advantageous for preventing not only constipation but also other gut‐related disorders. Although the precise mechanisms underpinning the relationship between DII and constipation remain elusive, mounting evidence suggests that inflammation and immune system dysfunction may play a critical role in the pathophysiology of constipation. Through comprehensive analysis of the majority of research data, we found a positive correlation between the severity of constipation and depression. However, in this study, we cannot observe significant differences during the trend analysis. We speculate that this might be attributed to our inclusion of only adult participants aged 20 years and above, while the adolescent depression population was not considered. Nevertheless, we still identified a significant correlation between constipation and depression.

Constipation can be caused by various factors, including a low‐fiber diet, dehydration, physical inactivity, certain medications, and medical conditions such as irritable bowel syndrome (IBS) and Parkinson's disease (Sundbøll et al., 2019). Studies in mice have also shown that a high‐fat diet leads to constipation by reducing colonic mucus (Mukai et al., 2020). In the elderly population, constipation is a common issue, especially in long‐term care settings, and can be caused by a variety of factors such as medication use, immobility, and inadequate fluid and fiber intake. It is important to identify the underlying cause of constipation in order to effectively improve it. In the study, there are several ways to prevent constipation, including maintaining a high‐fiber diet, drinking plenty of water, and engaging in regular physical activity. It is also important to establish a regular toileting routine and respond to bowel movement immediately. Avoiding or limiting certain foods, such as processed and fried foods, sugary drinks, and red meat, may also prevent constipation. In addition, for individuals taking medications causing constipation, such as opioids, it may be helpful to take a stool softener or laxative as directed by a healthcare professional. Providing advice on dietary consumption, exercise, and optimal position to defecate through education programs has also been found to be effective in preventing constipation (Rao et al., 2010). In summary, regardless of preventing or treating constipation, diet plays a key role.

The current study has some additional limitations. Firstly, the cross‐sectional design cannot establish a causal relationship between DII and constipation. Future longitudinal studies are needed to determine the temporal relationship between DII and constipation. Secondly, our assessment of DII relied on a single 24‐h dietary recall, which may not fully capture participants' long‐term dietary patterns. Third, the classification of constipation in this study was solely based on the weekly frequency of bowel movements, lacking clinical investigations into gastrointestinal diseases. Furthermore, medications, such as opioids and antipsychotics, are important factors that can cause constipation or diarrhea. The lack of sufficient sample size for physical inactivity data precluded its inclusion in this study. This study did not adjust for these factors as covariates in the model, which might have introduced bias.

## 5 CONCLUSIONS

Our study provides evidence of a potential association between DII and constipation, with higher DII scores associated with slightly increased odds of constipation. However, the relationship between DII and constipation may be more complex than previously thought and may vary depending on individual factors such as BMI and racial background. Further research is needed to establish the temporal relationship between DII and constipation and to investigate the underlying mechanisms of this association. The findings from this study have implications for public health interventions aimed at preventing and managing constipation, particularly among individuals with higher DII scores.

## AUTHOR CONTRIBUTIONS

**Bo Wang:** Conceptualization (equal). **Chunxiang Liu:** Conceptualization (equal). **Zheng Guo:** Data curation (equal). **Rui Li:** Writing – original draft (equal). **Yuchao Wang:** Writing – original draft (equal). **Caixia Dong:** Funding acquisition (equal). **Daqing Sun:** Funding acquisition (equal).

## FUNDING INFORMATION

This research was funded by the General Program of the National Natural Science Foundation of China (82070554 and 81770537); the Major Scientific and Technological Special Project for Public Health in Tianjin (21ZXGWSY00080); and the Tianjin Medical University General Hospital Clinical Research Program (22ZYYLCCG06).

## CONFLICT OF INTEREST STATEMENT

The authors declare no conflict of interest.

## Supporting information


Data S1

## Data Availability

Data described in the manuscript, code book, and analytic code will be made publicly and freely available without restriction at (https://www.cdc.gov/nchs/nhanes [accessed on 22 April 2023]).
